# Identification of an Immune-Related Prognostic Gene CLEC5A Based on Immune Microenvironment and Risk Modeling of Ovarian Cancer

**DOI:** 10.3389/fcell.2021.746932

**Published:** 2021-10-12

**Authors:** Jiacheng Shen, Tingwei Liu, Jia Lv, Shaohua Xu

**Affiliations:** ^1^Department of Gynecology, Shanghai First Maternity and Infant Hospital, School of Medicine, Tongji University, Shanghai, China; ^2^Department of Obstetrics and Gynecology, Shanghai Tenth People’s Hospital, School of Medicine, Tongji University, Shanghai, China

**Keywords:** ovarian cancer, immune microenvironment remodeling, CLEC5A, multi-omics, immunotherapy, prognostic modeling

## Abstract

**Objective:** To understand the immune characteristics of the ovarian cancer (OC) microenvironment and explore the differences of immune-related molecules and cells to establish an effective risk model and identify the molecules that significantly affected the immune response of OC, to help guide the diagnosis.

**Methods:** First, we calculate the TMEscore which reflects the immune microenvironment, and then analyze the molecular differences between patients with different immune characteristics, and determine the prognostic genes. Then, the risk model was established by least absolute shrinkage and selection operator (LASSO) analysis and combined with clinical data into a nomogram for diagnosis and prediction. Subsequently, the potential gene CLEC5A influencing the immune response of OC was identified from the prognostic genes by integrative immune-stromal analysis. The genomic alteration was explored based on copy number variant (CNV) and somatic mutation data.

**Results:** TMEscore was a prognostic indicator of OC. The prognosis of patients with high TMEscore was better. The risk model based on immune characteristics was a reliable index to predict the prognosis of patients, and the nomogram could comprehensively evaluate the prognosis of patients. Besides, CLEC5A was closely related to the abundance of immune cells, immune response, and the expression of immune checkpoints in the OC microenvironment. OC cells with high expression of CLEC5A increased the polarization of M2 macrophages. CLEC5A expression was significantly associated with TTN and CDK12 mutations and affected the copy number of tumor progression and immune-related genes.

**Conclusion:** The study of immune characteristics in the OC microenvironment and the risk model can reveal the factors affecting the prognosis and guide the clinical hierarchical treatment. CLEC5A can be used as a potential key gene affecting the immune microenvironment remodeling of OC, which provides a new perspective for improving the effect of OC immunotherapy.

## Introduction

Ovarian cancer (OC) is a kind of malignant tumor with a poor prognosis, and its mortality rate ranks second in gynecological cancer mortality (Bray et al., 2018). In recent years, with the improvement of the molecular basis of immune recognition and immune regulation of tumor cells, many kinds of cancer have been treated with immunotherapy. The immune microenvironment of tumor tissue can reflect the response rate to immunotherapy and chemotherapy (Rosenberg et al., 2016; Jiang et al., 2018). Understanding the immune regulatory network in the OC microenvironment will promote the effective use of immunotherapy.

The tumor microenvironment (TME) is a highly complex system. Tumor cells coexist with immune cells and non-immune cells and establish an interaction with them, which affects the tumor’s development and outcome. The immune cell components in TME are the basis for determining tumor fate and the cells’ ability to invade and metastasize (Melaiu et al., 2019; Vitale et al., 2019; Xiao et al., 2019). Their composition and interactions within the TME are closely related to clinical outcomes in cancer patients. Recent studies have shown that TME played a crucial role in cancer progression and treatment response, and advances in TME understanding have also contributed to the development of advanced cancer treatment methods (Jiang et al., 2018; Zeng et al., 2018). The composition of resident cell types in TME varies from different cancer patients. The number of CD8^+^ T cells, CD4^+^ T cells, macrophages, and tumor-associated fibroblasts in TME is associated with the clinical outcome of many malignant tumors, such as gastric cancer, urothelial cancer, lung cancer, and breast cancer (Fridman et al., 2017; Mantovani et al., 2017; Nishino et al., 2017; Mariathasan et al., 2018). There has been evidence that OC was an immunogenic tumor, and some studies have confirmed the prognostic value of the immune system in OC (Kandalaft et al., 2011). Therefore, understanding the differences of the immune microenvironment in OC may be an essential step in finding prognostic markers, stratifying patients before treatment, and prolonging life expectancy in OC patients.

In this study, we evaluated the TME characteristics of patients by calculating the TMEscore. Functional enrichment analysis showed that the TME signature could reflect the prognosis of OC patients. However, in the clinic, it is not realistic to calculate the TMEscore for each patient. Therefore, we integrate multiple differentially expressed prognostic genes based on the TMEscore into a model that can reflect the characteristics of TME, rather than using a single prognostic gene as an indicator, which will significantly improve the predictive value. We established a risk model by least absolute shrinkage and selection operator (LASSO) regression and further established a nomogram including risk score to predict the prognosis of OC patients. The risk model can not only reflect the TME characteristics but also predict the sensitivity of patients to platinum drugs and guide the clinical treatment of OC patients. In addition, we combined with the immune and stromal score, screened out the potential gene CLEC5A which was most likely to participate in the immune response of OC from these prognostic genes, and analyzed its expression in OC. Then, we further explored the effect of CLEC5A on M2 macrophage polarization through a co-culture experiment. This study opens up a new field of vision for the exploratory study of TME in OC and the prediction of the prognosis of OC patients.

## Materials and Methods

### Data Extraction and Processing

#### RNA-Seq Data

All the data in the current study were from the databases. The RNA-seq expression profile data and clinical information of OC patients were downloaded from the The Cancer Genome Atlas (TCGA) database.^1^ The overall parameters for downloading data were as follows: “Primary Site” was ovary, “Program” was TCGA, “Project” was TCGA-OV, and additional parameters for transcriptome data were the following: “Data Category” was transcriptome profiling, “Data Type” was gene expression quantification, “Experimental Strategy” was RNA-Seq, “Workflow Type” was HISeq-FPKM, and additional parameters for clinical data download were the following: “Data Category” was clinical, and “Data Type” was clinical supply. All samples were excluded for patients without survival information.

The RNA-seq expression profile data and clinical information of OC patients were downloaded from the International Cancer Genome Consortium (ICGC) OV-AU (Ovarian cancer-Australia) database,^2^ and samples were excluded for patients without survival information, which are used for subsequent validation. The statistical information of the preprocessed dataset is shown in Supplementary Table 1, and the detail clinical information of each patient with OC from these two databases is shown in Supplementary Tables 2, 3.

The transcriptome-level data of datasets GSE62873, GSE69207, and GSE146553 downloaded from the GEO database^3^ were used to verify the correlation between the expression level of CLEC5A and the proportion of immune cells after removing batch effects.

#### Noncoding RNA/Transcription Factor–mRNA Interaction Data

The noncoding RNA (ncRNA)–mRNA interaction and transcription factor (TF)-mRNA interaction data were downloaded from the RAID database (v2.0)^4^ and TRRUST v2 database.^5^ The interaction pairs of all ncRNAs/TFs and module genes were counted, and then the interaction pairs of each ncRNA/TFs and genes in or outside the module were counted.

#### Genomic Variation Data

The copy number variant (CNV) data of OC patients was downloaded from the TCGA database using the “TCGABIOLinks” package, and the data type was Masked Number Segment. The SNP6 GRCh38 Remapped Probeset File^6^ was used as the marker file, and the CNV interval was mapped to the corresponding gene. Somatic single-nucleotide variant (SSNV) data was the Mutect2 version in the TCGA database,^7^ which was the whole-exome sequencing data.

### Generation of TMEScore, Stromal Score, and Immune Score

Based on TCGA-OV RNA-seq data and clinical information, the “edger voom” algorithm was used to remove heteroscedasticity. Then, the proportions of 22 immune cell types in the OC were estimated by CIBERSORT (Newman et al., 2015). Next, we performed three unsupervised methods (consensus cluster, elbow method, and gap statistics) using the “factoextra” package and “ConsensusClusterPlus” package to explore the best number of clusters, and all three clustering methods were iterated 1,000 times to increase the stability of clustering. After the best number of clusters was selected, the k-means method was used to divide samples into two TME clusters and obtained the differentially expressed genes (DEGs) between two clusters using the “limma” package. We applied the consensus clustering method to explore sample classification again and used the chi-square test to compare the consistency of the two classifications (*p* = 2.089e-16). The random forest algorithm was performed using the “randomForest” package to reduce redundant genes and identify the core DEGs. The Cox regression model was used to classify the core genes and finally calculated the TMEscore of each patient using the formula as follows, which was similar to the algorithm mentioned in the previous study (Zeng et al., 2019).


$$\text{TMEscore}=\sum \text{l}\,o\,g_{2}\,\left(\text{X}+1\right)-\sum \text{l}\,o\,g_{2}\,\left(\text{Y}+1\right)$$

where X is the gene with the positive Cox coefficient, and Y means the gene with the negative Cox coefficient. The detailed procession for generating TMEscore is shown in Supplementary Figure 1.

The proportion of immune-stromal components of the TME which were represented in the form of the immune score and stromal score, respectively, in each OC sample was estimated utilizing the “estimate” package in R software.

### Identification of Differentially Expressed Genes Based on TMEscore, Stromal Score, and Immune Score

The OC samples were subdivided into the high- or low-score groups based on the TMEscore, stromal score, and immune score, respectively. The “limma” package was used to analyze the DEGs, and DEGs with filtering criteria of adj. *p*-values < 0.05 and | Fold Change| >1.5 were selected for further analysis.

### Survival and Functional Enrichment Analysis

Survival analysis refers to the method of analyzing and inferring the survival time of organisms or people according to the data obtained from experiments or surveys, exploring the relationship between survival time, outcome, and many influencing factors. Survival analysis of DEGs was performed by R using the “survminer” package. Genes with a log-rank *p*-value < 0.05 were considered as prognostic genes.

Functional enrichment analysis of related genes was performed using the “ClusterProfiler” package to identify significantly enriched Gene Ontology (GO) terms and Kyoto Encyclopedia of Genes and Genomes (KEGG) pathways. An adj. *p*-value < 0.05 was considered statistically significant. As for single-gene function analysis, the “GSEA” package was used in R software and the FDR *q*-value < 0.05 was set as the threshold to identify significantly important function terms.

### Construction of Protein–Protein Interaction and Pivot Network

The STRING database^8^ was used to build the protein–protein interaction (PPI) network of prognostic genes, and Cytoscape software (V3.6.0) was used to rebuild it. To identify closely connected modules in the network, the key module of the network was mined using the “MCODE” with criteria as MCODE score ≥3 and degree >10. The rank of hub genes was identified by “Cytohubba” in Cytoscape.

Pivot consists of participants that significantly regulate modules in OC tumorigenesis, including ncRNA and TF. The pivot is defined as follows: (1) the pivot has at least two interactions with the module gene and (2) the *p*-value of the significance analysis of the interaction between the pivot in which each module should be less than or equal to 0.05 (Wu et al., 2015). The hypergeometric test method was used for the significance analysis (see Supplementary Material).

### Construction of the Prognostic Risk Model and External Validation

The LASSO Cox regression model analysis was performed by the “glmnet” package to optimize prognostic genes in patients with OC (Simon et al., 2011). We resampled the dataset 1,000 times, and the best value of the penalty parameter λ was determined by 10-fold cross-validations. Meanwhile, variables with potential collinearity were excluded from the regression model (correlation < 0.5, variance inflation factor (VIF) <2). After core prognostic genes were selected, the multivariate Cox regression model was used to determine the regression coefficients (β) of each gene to establish a risk score formula:


The⁢Risk⁢Score=∑(β*expression⁢level)

All samples were subdivided into high- or low-risk according to the Risk Score of each sample. To evaluate the prognostic risk model’s predictive value, the time-dependent receiver operating characteristic (ROC) curve was performed using the “survivalROC” package. Finally, the risk model was validated in the ICGC OV-AU dataset.

### Independence of Risk Model and Construction of Nomogram

To determine the independence of the prognostic risk model in predicting the prognosis of OC patients, we performed a univariate and multivariate Cox regression analysis of several variables, which included risk score and calculated hazard ratio (HR), as well as 95% confidence intervals (CI). By combining all independent prognostic factors, a nomogram was constructed to evaluate the probability of 2-, 3-, 4-, and 5-year overall survival (OS) for patients with OC. The graph of prediction probability and observation rate was then drawn using the “rms” package, and the calibration curve of the prognostic model was graphically evaluated, which overlapped with the diagonal line, indicating that the model was utterly consistent. The decision curve analysis (DCA) was performed by the “rmda” package to test the clinical efficacy of the nomogram.

### Genomic Variation Analysis

Somatic variation data were retained in the mutation note format. Differentially mutated genes were identified by the “maftools” package with *p* < 0.05 as the significant threshold. The online tool GISTIC2.0^9^ was used to analyze CNV data. Specific amplifications and deletions of chromatin sites in each immune subtype were then selected for subsequent analysis.

### Clinical Specimens and Immunohistochemistry

The samples were collected from the Shanghai First Maternal and Infant Hospital and have obtained the informed consents (the ethical certification number: KS1748) with permission from the Medical Ethics Committee of Shanghai First Maternity and Infant Hospital. The diagnoses of acquired samples were all carefully certified and checked by experienced pathologists. The samples were fixed in 10% formalin, dehydrated, embedded and sectioned, and then immunohistochemically stained using VECTASTAIN Elite ABC Rabbit IgG Mini-PLUS Kit (PK-8501, Vector Laboratories, United States) with anti-CLEC5A antibody (ab203200, Abcam, Cambridge, United Kingdom) and hematoxylin (Wuhan Servicebio Technology Co., Ltd., Wuhan, China).

### Cell Culture and Co-culture Assay

The human leukemic cell line: THP-1 cell and human OC cell lines: SKOV3, HEY (American Type Culture Collection, Manassas, VA, United States), were cultured in RPMI 1640 medium (Wuhan Servicebio Technology Co., Ltd., Wuhan, China) containing 10% fetal bovine serum (Biological Industries, Beit HaEmek, Israel) and 1% penicillin/streptomycin (Wuhan Servicebio Technology Co., Ltd., Wuhan, China) at 37°C in a humidified atmosphere with 5% CO_2_. The THP-1 cells (5 × 10^4^ cells/100 μl) were seeded in a lower chamber and differentiated into macrophage by adding 100 ng/ml of phorbol 12-myristate-13-acetate (PMA, MCE, Chengdu, China) for 48 h. After THP-1 cells were differentiated into M0 macrophages, the M0 macrophages were treated with 20 ng/ml IL-4 (Peprotech, Rocky Hill, NJ, United States) plus 20 ng/ml IL-10 (Peprotech, Rocky Hill, NJ, United States) for 48 h to differentiate into M2 macrophages (Murray et al., 2014). The OC cells were transfected with NC or CLEC5A overexpression plasmid (ordered from Public Protein/Plasmid Library, China) using Lipofectamine 2000 Reagent (Invitrogen, United States) for 24 h, and the transfection efficiency was verified by real-time-qPCR (RT-qPCR). After transfection, the OC cells (3 × 10^4^ cells/100 μl) were then added to the upper insert (0.4 μm pore; 6-well Transwell, LABSELECT, Zhejiang, China) for co-culture for 24 h. In addition, we also transfected plasmids into M2 macrophages to detect the effect of CLEC5A on the polarization of M2 macrophages.

### RNA Extraction and Real-Time PCR Analysis

Total RNA from cells was extracted using the TRIzol reagent (TAKARA BIO INC., Japan) and then synthesized to complementary DNA (cDNA) using the 5 × ALL-IN-One RT Master Mix Kit (Applied Biological Materials Inc., Canada). TB Green Premix Ex Taq Kit (TAKARA BIO INC., Japan) was used for real-time PCR. The ΔΔCt values were normalized to GAPDH, and relative quantification of gene expression was compared to the NC group. The primers used in this study are listed in Supplementary Table 7 and synthesized by Sangon Biotech (Shanghai) Co., Ltd., (Shanghai, China).

### Statistical Analysis

Univariate survival analysis was performed using the Cox risk regression model, and a significance threshold of log-rank *p* < 0.05 was set to screen prognostic genes. The chi-square test was used to determine the significance of bias in the distribution of two clustering methods. The Wilcoxon rank test was used to determine significance in comparisons of two groups of continuous variables, the Kruskal–Wallis rank test was used for comparisons of more than two groups, and the Benjamini–Hochberg method was used to control the FDR. All the above analyses were performed using R version 3.6.0. Unless otherwise specified, ****p < 0.0001; ***p < 0.001; **p < 0.01; *p < 0.05; and ns, no significance.

## Results

### Quantification of Immune Infiltration and Identification of Molecular Characteristics in Patients With Ovarian Cancer

**FIGURE 1:**
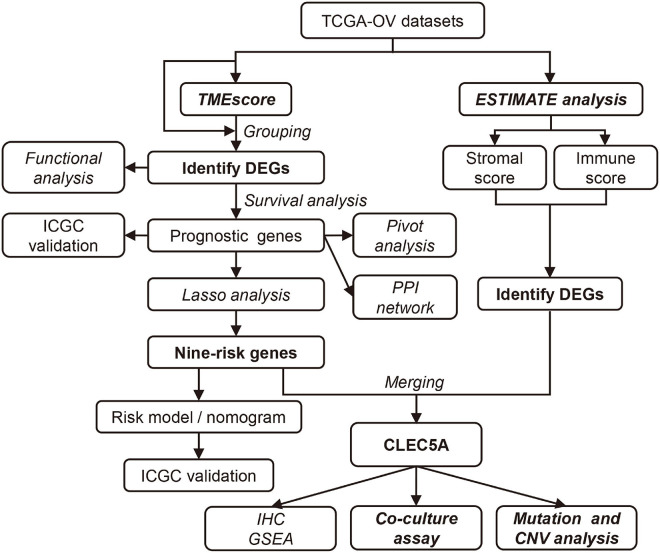
The workflow of study design.

**FIGURE 2:**
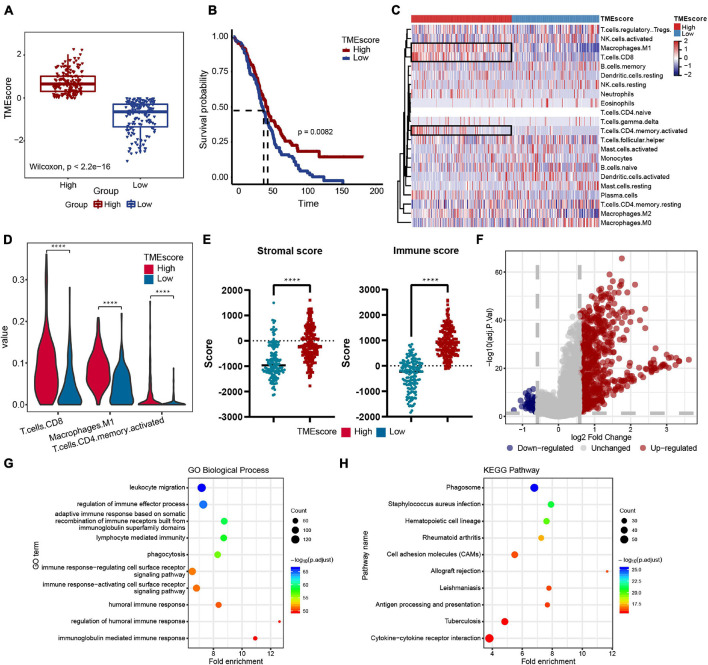
Immune features of the ovarian cancer (OC) microenvironment and identification of molecular characteristic. **(A)** TMEscore of samples in the TCGA-OV dataset (high group: 181 samples, low group: 158 samples). Wilcoxon’s test, *p* < 2.2e-16. **(B)** Overall survival (OS) Kaplan–Meier curves of TMEscore, log-rank test, *p* = 0.0082. **(C)** Heatmap of the 22 immune cell proportions in two TME group. **(D)** Violin plot of abundance of CD8^+^ T cells, M1 macrophages, and activated CD4^+^ memory T cells in the two groups. **(E)** Correlation between TMEscore and immune-stromal score. **(F)** Volcano plot of all differentially expressed genes (DEGs); the red dots represent upregulated genes, the blue dots represent downregulated genes, and the gray dots represent unchanged genes. **(G,H)** Genetic ontology (GO) biological process and Kyoto Encyclopedia of Genes and Genomes (KEGG) pathway enrichment analysis of DEGs; the fold enrichment represents the ratio of the gene ratio to background ratio. ****p < 0.0001.

The workflow design of this study is shown in Figure 1. By integrating the expression data and clinical data (OS >1 month, precise pathological stage), 339 OC samples were obtained for the generation of the TMEscore (Figure 2A). The survival analysis showed that a high TMEscore was significantly associated with a longer survival time (*p* = 0.0082) (Figure 2B). However, there was no statistical significance between TMEscore and pathological stage (*p* = 0.82, one of the OC patients was a stage I patient) (Supplementary Figure 2A). Analysis of the proportion of immune cells in tumor tissue of two groups divided by the TMEscore showed that the proportions of M1 macrophages, CD8^+^ T cells, and activated CD4^+^ memory T cells were increased in the high TMEscore group (Figures 2C,D), as well as the IFN-gamma related genes (Supplementary Figure 2B). The stromal score and immune score calculated by ESTIMATE were also significantly correlated with the TMEscore (Figure 2E). To identify the difference of the molecular level between the different degrees of immune infiltration, 747 genes were screened as DEGs for follow-up analysis according to the high- and low-TMEscore group, including 661 upregulated genes and 86 downregulated genes (Figure 2F and Supplementary Figure 2C). These DEGs were mainly enriched in immune-related functions and pathways, such as leukocyte migration, immune response, phagocytosis, cytokine–cytokine receptor interaction, and cell adhesion molecules (CAMs; Figures 2G,H).

### Identification of Prognostic Genes and Construction of the Protein–Protein Interaction Network

**FIGURE 3:**
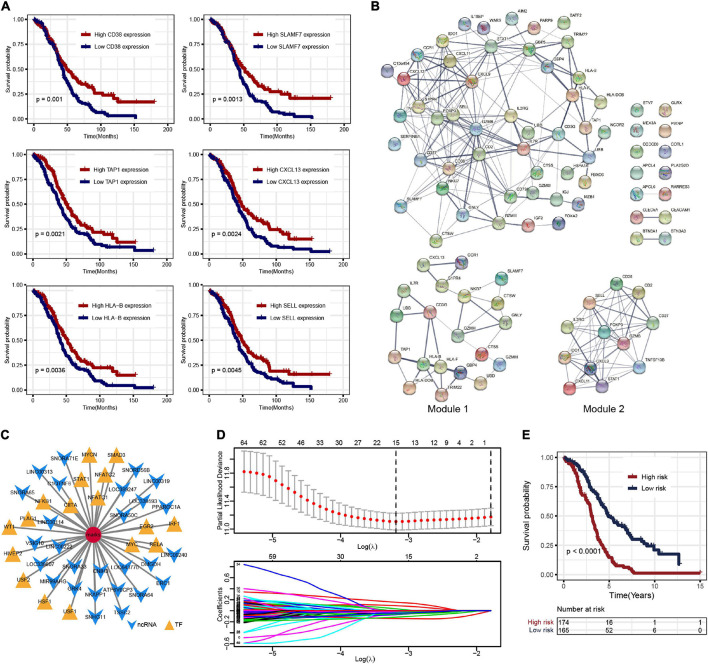
**(A)** Overall survival Kaplan–Meier curves of DEGs (show top six genes). **(B)** The protein–protein interaction (PPI) network of prognostic genes and the two key modules. **(C)** Noncoding RNA (ncRNA) and transcription factor (TF)-pivot of prognostic genes; red circular mark represents 83 prognostic genes, yellow arrows represent ncRNAs, and green triangles represent TF. **(D)** Tenfold cross-validation for tuning parameter selection in the least absolute shrinkage and selection operator (LASSO) model. **(E)** The K–M survival curves show the OS based on the high- and low-risk patients.

To further explore the prognosis-related molecular characteristics, 83 prognostic genes associated with OS (*p* < 0.05) were screened and the top six significant genes are shown in Figure 3A. These 83 prognostic genes still showed significant enrichment of immune-related functions or pathways such as lymphocyte-mediated immunity, B cell-mediated immunity, cytokine–cytokine receptor interaction, and CAMs (Supplementary Figure 2D). External validation of these prognostic genes was performed in the ICGC OV-AU dataset, in which 58 of the 83 genes were expressed. Further survival analysis showed that seven genes were identified as consistent with the results in the TCGA-OV dataset (Supplementary Figure 2E). Some genes have been validated in OC, so these genes were likely prognostic biomarkers in the OC immune microenvironment (Leffers et al., 2009; Ignacio et al., 2018; Nowak et al., 2018). To better understand the interactions among prognostic genes, all prognostic genes were analyzed in the STRING database to construct the PPI network (Figure 3B). Further analysis using the “MCODE” found two modules (Figure 3B). The module genes were significantly associated with immune regulation, such as CXCL9, CXCL11, CXCL13, CCR1, and CD27, as well as NAD metabolism-related genes, including IDO1 and CD38.

### Construction of the Pivot Network of Noncoding RNA and Transcription Factor

Based on the 51,913 pairs of ncRNA–mRNA interaction from the RAID 2.0 database as the background of interaction, 27 ncRNAs regulating the prognostic genes were screened, including several ncRNAs associated with OC, such as SNORA64, ATP6V0CP3, SNHG11, and PPARGC1A (Supplementary Table 4). The TFs regulating the prognostic genes were screened based on the 9,396 pairs of TF–mRNA interaction in the TRRUST v2 database. There were 17 TFs that regulate prognostic genes, including several TFs associated with OC, such as *NFKB1*, *MYC*, *IRF1*, and *STAT1* (Supplementary Table 5). The interactions among prognostic genes, ncRNAs, and TFs were visualized by Cytoscape, as shown in Figure 3C.

### Construction of Risk Model to Predict Prognosis in Ovarian Cancer Patients and External Validation

**FIGURE 4:**
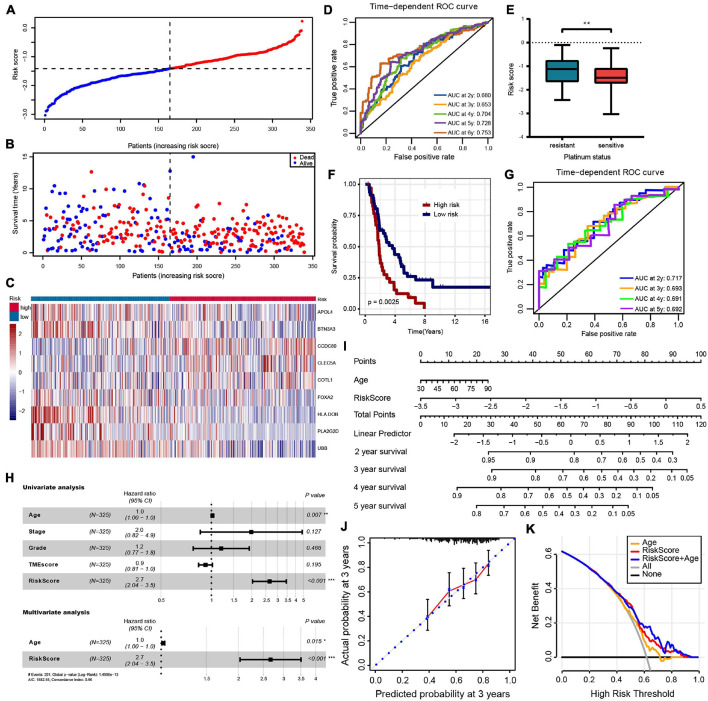
**(A–C)** The risk score distribution, patients’ survival status and gene expression profiles are displayed; the black-dotted line divided patients into high- and low-risk groups. **(D)** Time-dependent receiver operating characteristic (ROC) curve analysis of survival prediction by the prognostic model. **(E)** The risk score of platinum-resistant patients with OC is significantly higher than that of platinum-sensitive patients. **(F,G)** OS K–M curves and time-dependent ROC curves for the prognostic model in the ICGC OV-AU cohort. **(H)** Univariate and multivariate associations of the prognostic model and clinical characteristics with OS. **(I)** The nomogram is applied by adding up the points identified on the point scale for each variable. The total points projected on the bottom scales indicate the probability of 2-, 3- 4-, and 5-year OS. **(J)** The calibration curve for predicting 3-year OS for patients with OC; the *Y*-axis represents actual survival, as measured by K–M analysis, and the *X*-axis represents the prognostic model-predicted survival. **(K)** The net benefit (*Y*-axis) of age, risk model, and the nomogram; the black solid line represents the assumption that all patients survived, the gray solid line represents the assumption that no patients survived, and the orange, red, or blue solid lines represent the age, risk model, and nomograms. ***p < 0.001; **p < 0.01; *p < 0.05.

Least absolute shrinkage and selection operator regression analysis was performed with 10-fold cross-validation to further select the genes from 83 prognostic genes (Figure 3D). After excluding intergenic collinearity by correlation coefficient and VIF, nine genes were eventually selected to construct a risk model (Supplementary Figure 3A). According to the formula Risk Score = (−0.0780* APOL4) + (−0.2073* BTN3A3) + (0.1201* CCDC80) + (0.1958* CLEC5A) + (0.1917* COTL1) + (−0.1102* FOXA2) + (−0.2001* HLA-DOB) + (−0.3214* PLA2G2D) + (−0.1822* UBB), each patient’s risk score was calculated. Then, 339 patients with risk scores were classified into the high- or low-risk group (Figures 4A–C), and the K–M survival curves of the two groups had a significant difference (*p* < 0.0001) (Figure 3E). The prognostic ability of the model was evaluated by ROC. As a result, the areas under the curve (AUC) of the risk model were 0.680, 0.653, 0.704, 0.728, and 0.753 for the 2-, 3-, 4-, 5-, and 6-year survival times, indicating that the prediction of the risk model had a good performance and the longer patients survive the better the fitness (Figure 4D). Further analysis of the relationship between the risk score and the response status of OC patients to platinum drugs found that the risk scores of platinum-resistant patients were significantly higher than those of platinum-sensitive patients (*p* = 0.0032) (Figure 4E).

For external validation in ICGC databases, each patient was scored using the same risk score formula and grouped by the same cutoff value. Consistent with the result of the TCGA-OV cohort, the OS of patients in the low-risk group was significantly higher than that in the high-risk group (*p* = 0.012) (Figure 4F). The ROC analysis showed that AUCs of the prognostic model were 0.717, 0.693, 0.691, and 0.692 at 2-, 3-, 4-, and 5- years, respectively, suggesting that this risk model was able to predict OS in OC patients (Figure 4G).

### Independent Predictive Value of Risk Model and Nomogram Constructing

To evaluate the independent predictive value of the risk model, we performed univariate and multivariate Cox regression analyses on 325 OC patients with complete clinical data in the TCGA-OV dataset. We reassessed the risk model on this patient subset, showing still good benefits (Supplementary Figure 2F). Univariate Cox regression analysis showed that age and prognostic risk models had a certain predictive value for prognosis. In contrast, pathological stage, histological grade, and TMEscore were not independent prognostic factors (Figure 4H and Supplementary Table 6). We incorporated the age and risk model into multivariate Cox regression analysis, and the results showed that age and risk model were independent prognostic elements associated with OS (Figure 4H and Supplementary Table 6). To create a method to predict the individual survival likelihood of OC patients to guide clinical diagnosis and treatment, we subsequently developed a nomogram including two independent prognostic factors (the age and risk score) to predict the probability of the 2-, 3-, 4-, and 5-year OS in the TCGA-OV cohort (Figure 4I). Calibration curves used to visualize the nomogram’s performance showed that the nomogram had an excellent predictive value (Figure 4J and Supplementary Figure 3B). The DCA curve was used to test the clinical efficacy, and the results showed that the net benefit rate of the nomogram-combined risk model and age increased significantly (Figure 4K). Moreover, the nomogram (age + risk score) also has a good predictive value in the ICGC dataset (Supplementary Figure 3C).

### CLEC5A Is the Key Prognostic Gene of Ovarian Cancer Associated With Immunity

**FIGURE 5:**
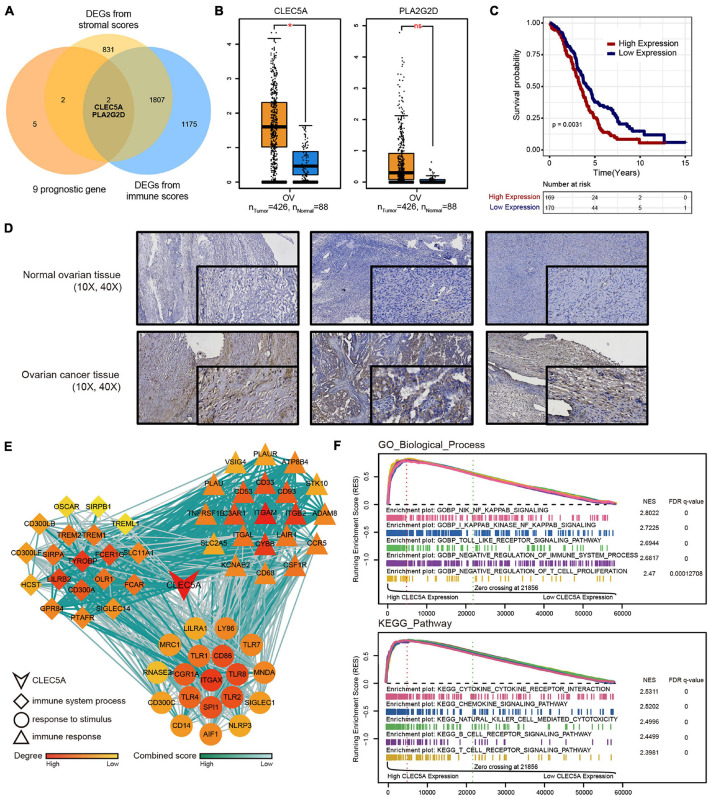
**(A)** Venn diagram of DEGs from TMEscore and immune-stromal score. **(B)** Expression difference of CLEC5A and PLA2G2D in normal ovarian tissue and OC. **(C)** OS Kaplan–Meier curves of CLEC5A. **(D)** Expression of CLEC5A in normal ovarian tissue and OC (immunohistochemical staining). **(E)** The functions of three groups of CLEC5A-related genes. **(F)** Single-gene GSEA analysis of CLEC5A (GO-BP and KEGG pathway).*, *p* < 0.05; ns, no significance.

To find the key genes that affect the immune microenvironment of OC in the prognostic genes, we combined the stromal score and immune score obtained by ESTIMATE analysis based on TMEscore and finally screened CLEC5A and PLA2G2D (Figure 5A). There were differences in the expression level of CLCE5A between tumor tissues and normal tissues, but there was no significant difference between PLA2G2D (Figure 5B), and CLEC5A significantly affected the survival of patients (Figure 5C), indicating that CLEC5A was more suitable as a prognostic gene than PLA2G2D. There was no significant correlation between the expression of CLEC5A and other clinical features of OC, such as FIGO stage and histological grade (Supplementary Figure 4A). Immunohistochemistry showed that the expression of CLEC5A in OC tissue was significantly higher than that in normal ovarian tissue (Figure 5D). To further analyze the role of CLEC5A in the immune microenvironment of OC, we first divided CLEC5A-related genes into three groups. The functions of these three groups of genes were significantly related to the immune response, the process of the immune system, and the response to stimulation (Figure 5E). Then, we performed the single-gene GSEA analysis of CLEC5A, and the results showed that CLEC5A significantly regulated various immune cell pathways; in addition, it was also significantly related to the NF-κB pathway, Toll-like receptor pathway, cytokine, and chemokine pathway (Figure 5F).

### CLEC5A Has the Potential to Remodel the Tumor Microenvironment of Ovarian Cancer and Influence the Immune Response

**FIGURE 6:**
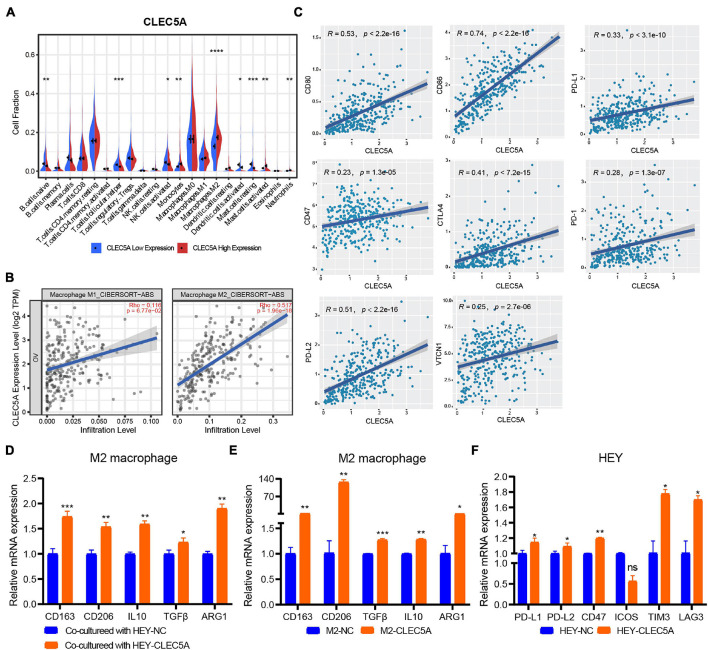
**(A)** Correlation analysis of CLEC5A expression and immune cell components in the TME of OC (TCGA-OV dataset). **(B)** Correlation analysis between the expression of CLEC5A and the infiltration of M1 and M2 macrophages in TME of OC. **(C)** Correlation analysis of CLEC5A expression and immune checkpoint gene. **(D)** When the M2 macrophages were co-culture with OC cell line HEY overexpressing CLEC5A, the M2 polarization level of macrophages increased. **(E)** After overexpression of CLEC5A in M2 macrophages, the M2 polarization level increased. **(F)** The expression of immune checkpoint genes PD-L1, PD-L1, CD47, TIM3, and LAG3 increased in OC cell line HEY overexpressing CLEC5A. ****p < 0.0001; ***p < 0.001; **p < 0.01; *p < 0.05; ns, no significance.

**FIGURE 7:**
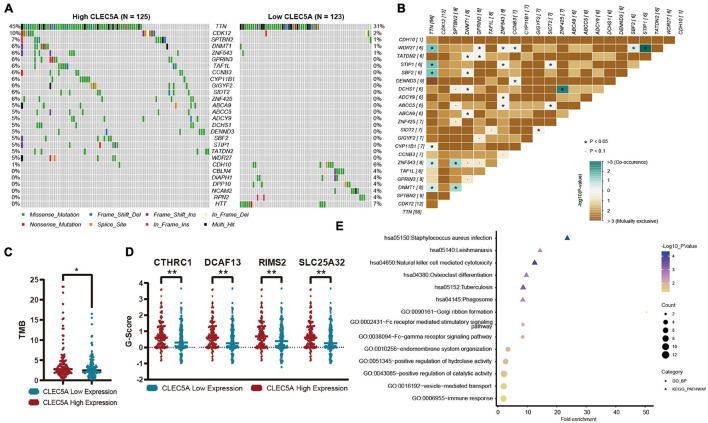
**(A)** Mutation genes with significant difference between high- and low-expression CLEC5A groups. **(B)** Analysis of co-mutation and exclusive mutations in the CLEC5A overexpression group. **(C)** The difference of tumor mutational burden (TMB) levels in high- and low-expression CLEC5A groups. **(D)** Genes with significantly increased copy number variation in CLEC5A overexpression group. **(E)** Functional analysis of genes with decreased copy number variation in CLEC5A overexpression group. **p < 0.01; *p < 0.05.

According to the expression level of CLEC5A, the patients were divided into high- and low-expression groups. The relationship between the expression level of CLEC5A and immune cells in the immune microenvironment was further analyzed. It was found that the abundance of B cells naïve, T cells follicular helper, and NK cells activated in TME of patients with high CLEC5A expression was significantly lower than that of patients with low expression, while the abundance of M2 macrophages was much higher than that of patients with low expression (Figures 6A,B). The analysis of three datasets (GSE62873, GSE69207, and GSE146553) in the GEO database also showed the correlation between CLEC5A expression and M2 macrophages (Supplementary Figure 4B). To verify the correlation between CLEC5A and M2 macrophages, we carried out the co-culture assay of OC cells and M2 macrophages. Co-culture assay showed that OC cells overexpressing CLEC5A could enhance the M2 polarization of M2 macrophages (Figure 6D and Supplementary Figure 4C). Moreover, overexpression of CLEC5A in M2 macrophages can also enhance its own M2 polarization (Figure 6E), as in a previous study (Tong et al., 2020). The correlation analysis between CLEC5A and immune checkpoint gene suggested that CLEC5A was positively correlated with CD80, CD86, PD-L1, CTLA4, CD47, and PD-L2 (Figure 6C). RT-PCR analysis showed that the expression of immune checkpoints such as PD-L1, PD-L2, and CD47 in OC cells overexpressing CLEC5A was also increased (Figure 6F and Supplementary Figure 4D), suggesting that OC patients with high expression of CLEC5A may have a better therapeutic effect on immune checkpoint inhibitors. Mutation analysis showed that the tumor mutational burden (TMB) was significantly increased in OC patients with high expression of CLEC5A (Figure 7C). Moreover, the mutations of TTN, CDK12, TAF1L, and DNMT1 genes in these patients were significantly higher than those in patients with low expression of CLEC5A, and the co-mutations occurred frequently (Figures 7A,B). Through CNV analysis, we further found that the copy number of CTHRC1, DCAF13, RIMS2, and SLC25A32 was significantly increased in patients with high CLEC5A expression (Figure 7D). Most of these genes were proved to be closely related to the occurrence and development of tumors in previous studies (Wang K. et al., 2019; Ding et al., 2020; Hill et al., 2020; Liu et al., 2020; Mei et al., 2020; Santoro et al., 2020). In the high CLEC5A group, the low copy number genes were significantly related to NK cell killing, Fc-gamma signaling pathway, and immune response (Figure 7E).

## Discussion

Ovarian cancer is common in women and is considered one of the most lethal malignancies. The high mortality of OC is usually due to the failure of early diagnosis. Although the current treatment plan is reasonably effective in the early stages, most patients ultimately recur and eventually develop chemoresistance. An increasing number of studies have shown that immune cell infiltration in the TME plays a vital role in regulating the occurrence and therapeutic efficacy of OC (Au et al., 2017; Curtis et al., 2018; Singel et al., 2019). In recent years, several immunoscore-based models have been reported to quantify the immune environment in tumor tissue and provide a statistically powerful indicator for the prognosis of patients with various solid tumors (Galon et al., 2012, 2014; Jiang et al., 2018). Earlier studies by Zeng et al. (2019) suggested that the TMEscore had good value in predicting the survival of patients with gastric cancer and guiding more effective immunotherapy strategies. Hence, we assessed the TME in OC patients using the TMEscore in this study. Our findings suggested that the assessment of OC immune status by the TMEscore provided a predictor of survival in OC patients. The TME-relevant genes highly expressed in the high TMEscore group significantly related to immune cell activation and proliferation, response to IFN-γ, and cellular killer.

Previous studies have shown that M1 tumor-associated macrophages (TAMs), CD8^+^ T cells, and CD4^+^ T cells played a crucial role in controlling tumor metastasis and prognosis (Borst et al., 2018; Yang et al., 2018; van der Leun et al., 2020). The present study supported the previous views that our results showed a significant increase in the proportion of M1 macrophages, CD8^+^ T cells, and CD4^+^ T cells in the high TMEscore group patients with OC, and the survival rates of these patients increased significantly. Besides, the chemokines secreted by the M1 macrophages and the cytolytic molecules associated with cell killing were highly expressed in the high TMEscore group. We further analyzed the PPI networks and found a close relationship between these genes involved in antitumor immunity. Several ncRNAs (SNORA64, ATP6V0CP3, SNHG11, PPARGC1A) and TFs (*NFKB1*, *IRF1*, and *STAT1*) regulating prognostic genes found in pivot analysis were associated with OC (Permuth-Wey et al., 2011; Huo et al., 2013; Pavan et al., 2013; Dong et al., 2014; Au et al., 2016; Zhao and Fan, 2019). Taken together, these results demonstrated that the TMEscore could reflect the immune microenvironment of the tumor and the prognosis of patients with OC.

Although a single biomarker for predicting prognosis has been widely reported, a single gene cannot reflect the complexity of TME well. It is necessary to establish a multi-gene prognostic risk model (Long et al., 2018; Wang R. et al., 2019). In our study, we combined the TMEscore reflecting the prognosis with LASSO for the first time to develop a risk model that reflected both TME characteristics and OS in OC patients. We identified nine genes to construct the risk model which had a good performance for OS prediction according to the survival and ROC analysis. Besides, the significant consistency between the risk score groups and the TMEscore groups (χ^2^ contingency tests, *p* = 0.001) demonstrates that the risk model can reflect the TME signature of patients with OC to some extent. The relationship between risk score and platinum response indicated that this risk model can be used to predict the sensitivity to platinum drugs in patients with OC and guide the clinical treatment. In addition, we confirmed that the risk score was independent of other relative factors such as pathological stage, histological grade, and TMEscore in patients with OC. Then, the nomogram based on age and risk score was constructed to precisely predict the survival probability in OC patients. The calibration curves and DCA curve showed that the actual survival was closely related to predicted survival, indicating that the nomogram could predict the OS of patients with OC well. Therefore, clinicians can offer individualized treatment for OC patients according to their nomogram predictions.

To select the genes that are most closely related to tumor immunity from these key prognostic genes, we further combined with the DEGs based on ESTIMATE analysis to screen CLEC5A as the key prognostic gene. Previous studies on CLEC5A are mostly related to the immune response caused by microbial viruses, and most of the studies related to tumors focus on glioblastoma, but the research in OC has been a vacancy (Fan et al., 2019; Sung et al., 2019; Tang et al., 2019). Our study found that compared with normal ovarian tissue, the expression of CLEC5A in OC increased significantly, and the higher the expression of CLEC5A, the lower the survival rate of patients. Previous studies have shown that CLEC5A expression in glioma patients was significantly correlated with immunosuppression and survival rate and could be used as a marker of M2 macrophages (Tong et al., 2020). The same pattern was also found in OC that M2 macrophage infiltration abundance was significantly increased in OC patients with high expression of CLEC5A. When OC cells overexpressing CLEC5A were co-cultured with M2 macrophages, the polarization of M2 macrophages increased. Moreover, M2 macrophages overexpressing CLEC5A also showed enhanced M2 polarization. The expression of immune checkpoint genes in OC patients was also closely related to CLEC5A, which indicates that CLEC5A may affect the response of OC patients to immune drugs. The functional analysis of CLEC5A showed that it was closely related to various immune cell pathways and the NF-κB pathway, which provides some clues for the follow-up mechanism research. The TMB of patients with high expression of CLEC5A was increased, and there were high-frequency mutations of TTN, CDK12, TAF1L, and DNMT1, which have been proved to be closely related to the occurrence and development of tumors in previous studies (Tien et al., 2017; Zhong et al., 2019; Yang et al., 2020). In addition, in patients with high expression of CLEC5A, sites with reduced genome copy number significantly affected the immune response pathway. These results suggest that CLEC5A could be used as a potential prognostic marker for OC patients.

In conclusion, TMEscore is an indicator of OC prognosis, and patients with a high TMEscore often have a good prognosis. The risk model based on the prognostic genes analyzed by TME signature is a dependable indicator for predicting the OS of OC patients, and the nomogram containing prognostic models can comprehensively evaluate the prognosis of patients. In addition, we confirmed for the first time that CLEC5A was a potential factor affecting the immune microenvironment of OC, and more research is needed to explore its importance in microenvironment remodeling, to further improve the effect of OC immunotherapy.

## Data Availability Statement

The RNA-seq expression profile data and clinical information of OC patients were downloaded from the TCGA database (https://tcga-data.nci.nih.gov/tcga/).

## Ethics Statement

The studies involving human participants were reviewed and approved by Medical Ethics Committee of Shanghai First Maternity and Infant Hospital (the ethical certification number: KS1748). The patients/participants provided their written informed consent to participate in this study.

## Author Contributions

JS and TL collected and assembled the data, performed the data analysis, and wrote the manuscript. JL performed the immunohistochemistry. SX provided the conception and helped with the manuscript and data review finally. All authors contributed to the article and approved the submitted version.

## Conflict of Interest

The authors declare that the research was conducted in the absence of any commercial or financial relationships that could be construed as a potential conflict of interest.

## Publisher’s Note

All claims expressed in this article are solely those of the authors and do not necessarily represent those of their affiliated organizations, or those of the publisher, the editors and the reviewers. Any product that may be evaluated in this article, or claim that may be made by its manufacturer, is not guaranteed or endorsed by the publisher.

## Footnotes

1
https://tcga-data.nci.nih.gov/tcga/

2
https://icgc.org/

3
https://www.ncbi.nlm.nih.gov/geo/

4
http://www.rna-society.org/raid2/

5
http://www.grnpedia.org/trrust/

6
https://gdc.cancer.gov/about-data/gdc-data-processing/gdc-reference-files

7
https://www.cancer.gov/about-nci/organization/ccg/research/structural-genomics/tcga

8
https://string-db.org/

9
https://cloud.genepattern.org/

## Abbreviations

TCGA: The Cancer Genome Atlas
ICGC: International Cancer Genome Consortium
OC: ovarian cancer
TME: tumor microenvironment
DEG: differentially expressed gene
GO: Genetic ontology
KEGG: Kyoto Encyclopedia of Genes and Genomes
OS: overall survival
ROC: receiver operating characteristic
AUC: area under the curve
LASSO: least absolute shrinkage and selection operator
TAM: tumor-associated macrophages
CAM: cell adhesion molecules
TF: transcription factor
ncRNA: noncoding RNA
DCA: decision curve analysis
HR: hazard ratio
CI: confidence interval
PPI: protein–protein interaction
VIF: variance inflation factor
TMB: tumor mutational burden.
