# Supplementary material for: Identification of an Immune-Related Prognostic Gene CLEC5A Based on Immune Microenvironment and Risk Modeling of Ovarian Cancer
Source: Front Cell Dev Biol. 2021 Oct 12;9:746932. doi: 10.3389/fcell.2021.746932 (PMC8547616; doi:10.3389/fcell.2021.746932)
Supplement: Supplementary file 1 [file Table_1.docx]

Supplementary Material

# Supplementary Material

**The hypergeometric test method used in Pivot analysis:**

$$\boldsymbol{p}\left( \boldsymbol{k} \right)\boldsymbol{=P}\left( \boldsymbol{X=k} \right)\boldsymbol{=1-}\sum_{\boldsymbol{M-1}}^{\boldsymbol{k=0}} \frac{\left( \begin{matrix} \boldsymbol{M} \\ \boldsymbol{k} \end{matrix} \right)\left( \begin{matrix} \boldsymbol{N-M} \\ \boldsymbol{n-k} \end{matrix} \right)}{\left( \begin{matrix} \boldsymbol{N} \\ \boldsymbol{n} \end{matrix} \right)}$$

Where N is the total number of the interaction pairs, n represents the number of interaction pairs of a certain ncRNA/TF. M is the total number of interaction pairs of genes belonging to the module with ncRNA/TF, while k represents the interaction pairs of genes in the modules with a certain ncRNA/TF.

# Supplementary Figures and Tables

## Supplementary Figures


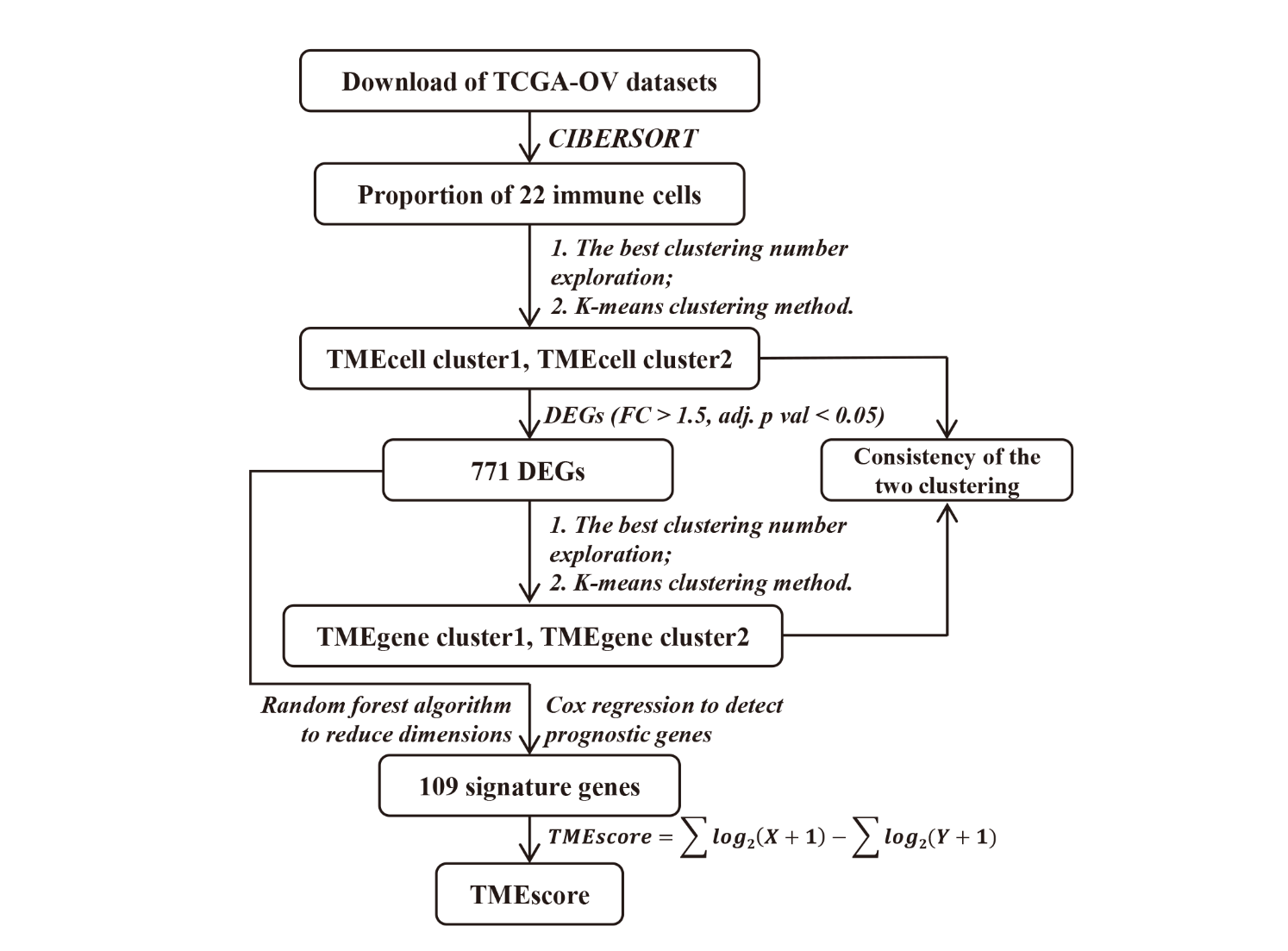


**Supplementary Figure 1.** The workflow of generating the TMEscore.


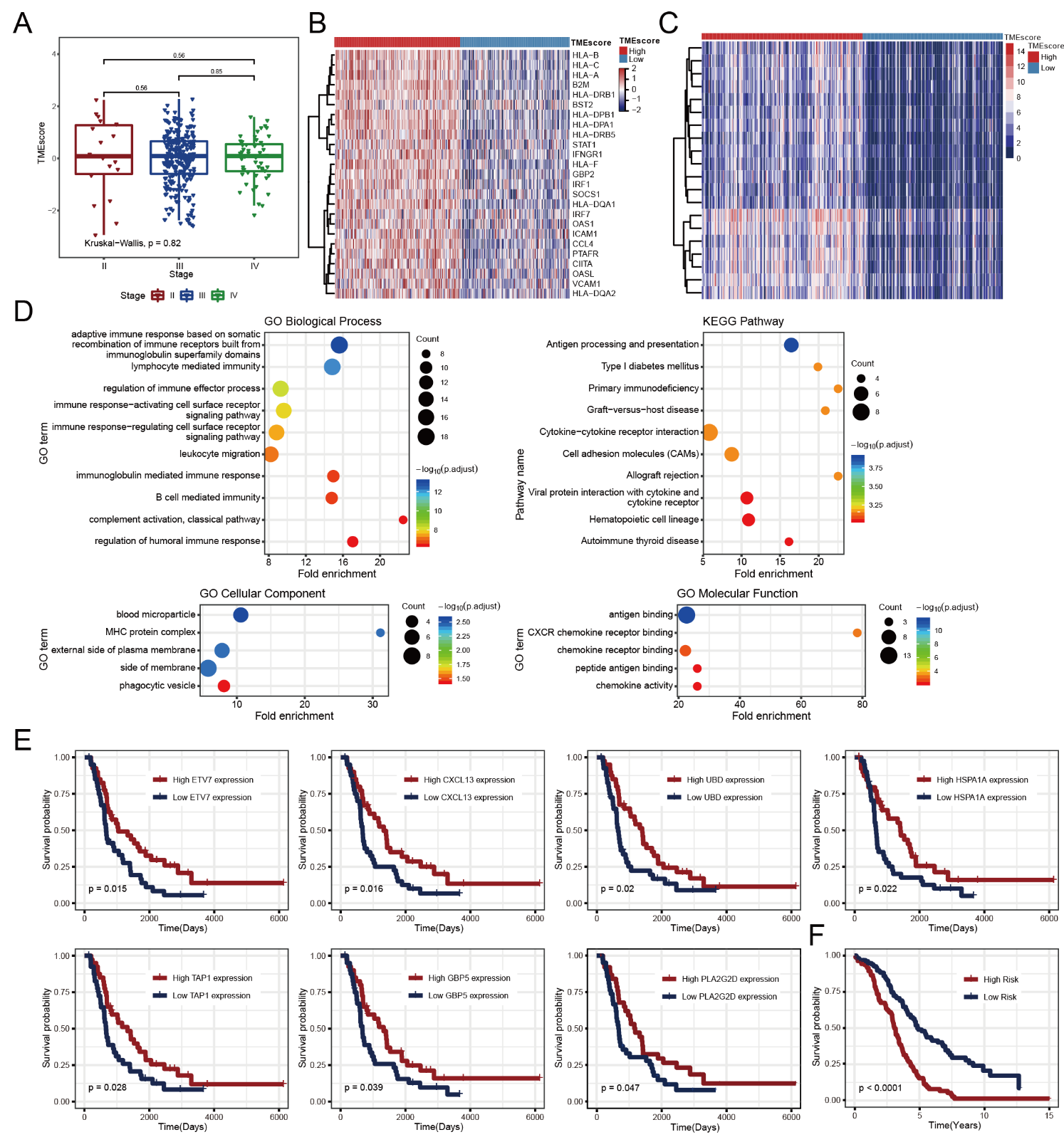


**Supplementary Figure 2.** **(A)** Association between TME score and ovarian cancer stage, Kruskal-Wallis test, p = 0.82. **(B)** Heatmap of the IFN-related genes expression in two TME groups. **(C)** Top 20 genes identified as the most differentially expressed in high TME score group compared with low TME score group. **(D)** Functional and pathway enrichment analysis of prognostic genes. **(E)** OS Kaplan-Meier curves of prognostic genes in ICGC OV-AU dataset. **(F)** Re-evaluation of the risk model on the subset of training data.


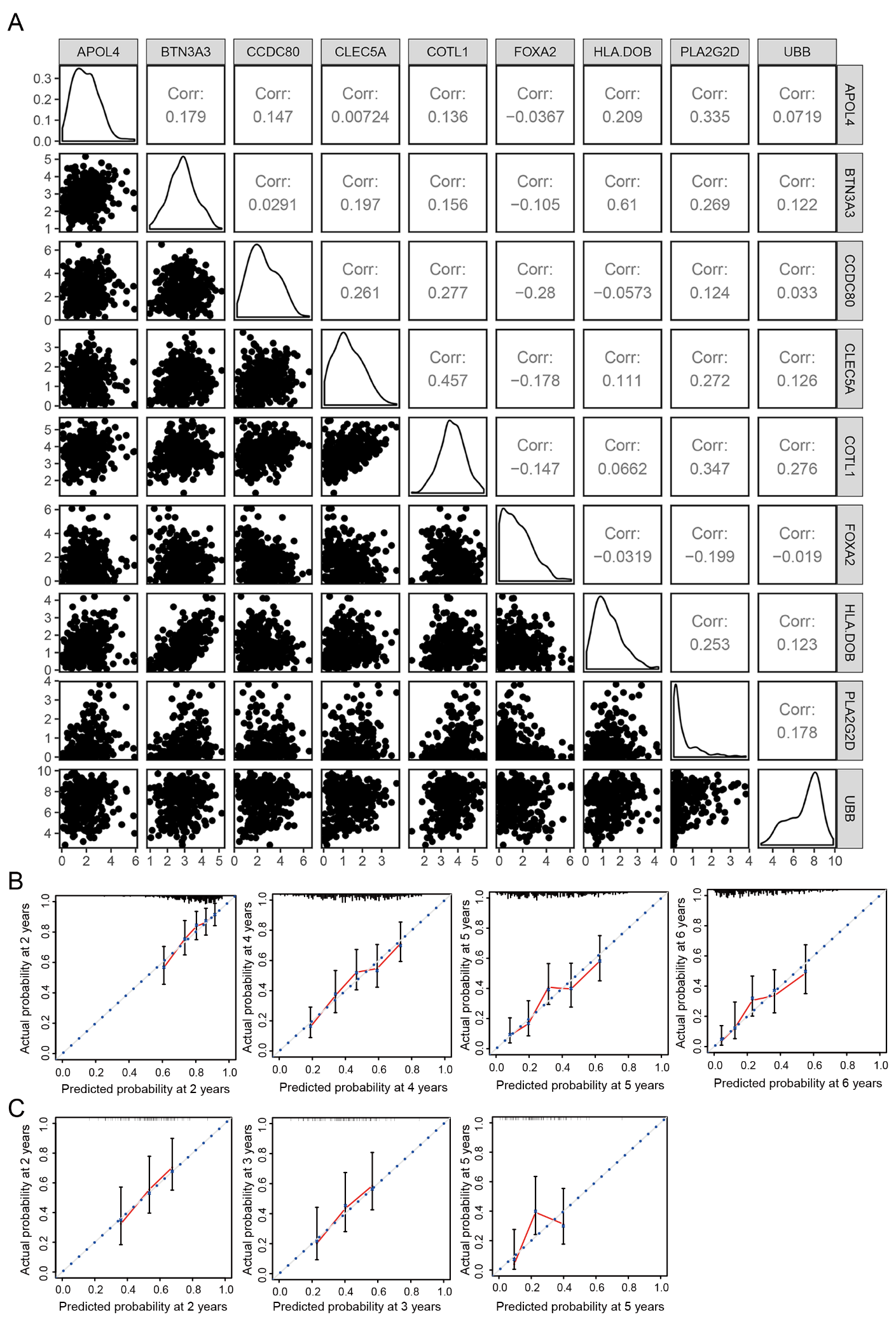


**Supplementary Figure 3.** **(A)** The correlation plot of nine genes selected from the prognostic genes. **(B)** The calibration curve for predicting 2-, 4-, 5-, and 6-year OS for patients with OC in TCGA dataset, the Y-axis represents actual survival, as measured by K-M analysis, and the X-axis represents the prognostic model-predicted survival. **(C)** The calibration curve for predicting 2-, 3-, and 5-year OS for patients with OC in ICGC dataset, the Y-axis represents actual survival, as measured by K-M analysis, and the X-axis represents the prognostic model-predicted survival.


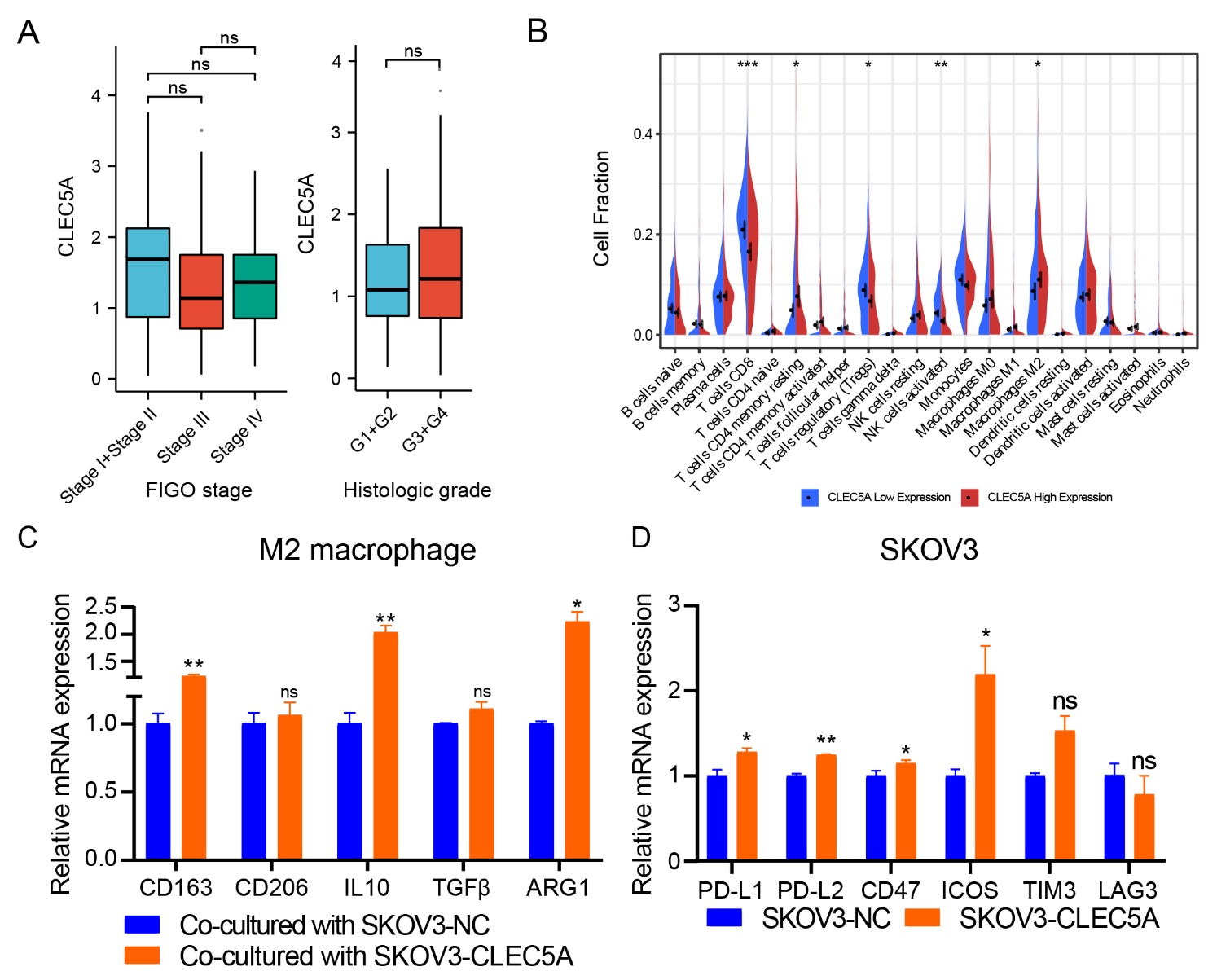


**Supplementary Figure 4. (A)** The expression of CLEC5A in OC had no significant correlation with its FIGO stage and histological grade. **(B)** Correlation analysis of CLEC5A expression and immune cell components in TME of OC (GEO datasets: GSE62873, GSE69207, GSE146553). **(C)** When the M2 macrophages were co-cultured with OC cell line SKOV3 overexpressing CLEC5A, the M2 polarization level of macrophages increased. **(D)** The expression of immune checkpoint genes PD-L1, PD-L2, CD47 and ICOS increased in OC cell line SKOV3 overexpressing CLEC5A.

## Supplementary Tables

Please see the ‘Supplementary Table.xlsx’ file for details.
