# Supplementary figures and images for: Identification of an Immune-Related Prognostic Gene CLEC5A Based on Immune Microenvironment and Risk Modeling of Ovarian Cancer
Source: Front Cell Dev Biol. 2021 Oct 12;9:746932. doi: 10.3389/fcell.2021.746932 (PMC8547616; doi:10.3389/fcell.2021.746932)

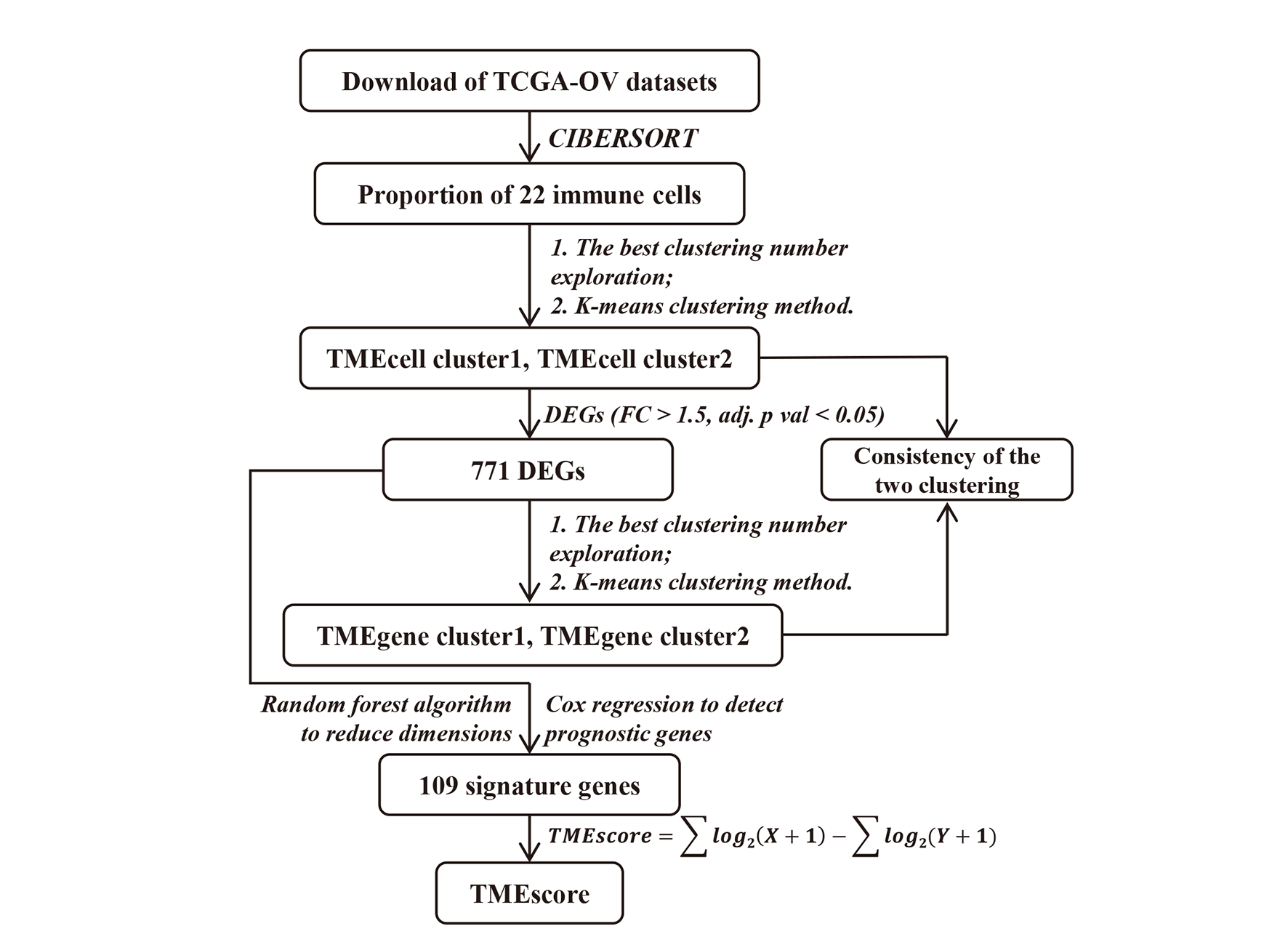

Supplement: Supplementary file 3 [file Image_1.TIF]

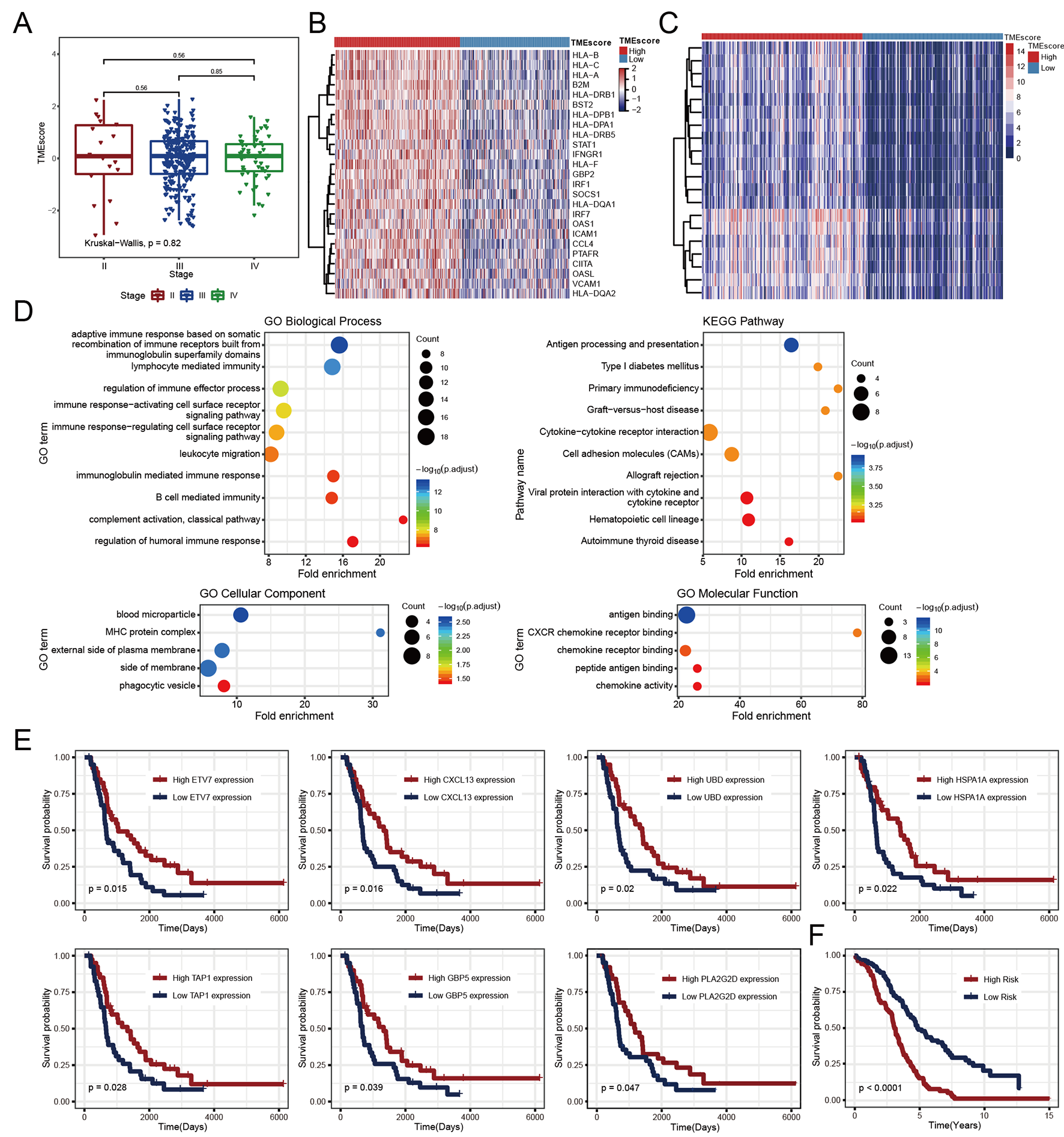

Supplement: Supplementary file 4 [file Image_2.TIF]

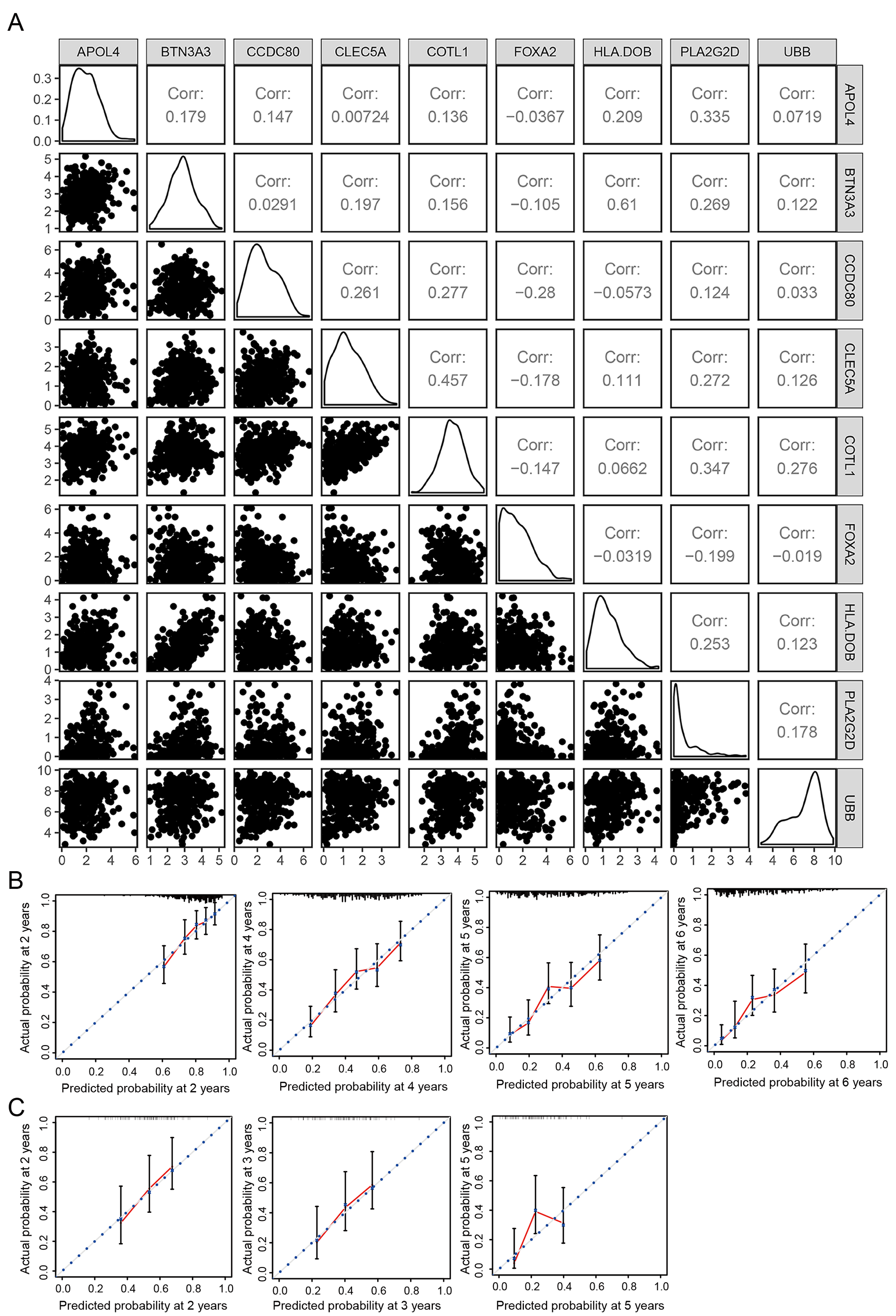

Supplement: Supplementary file 5 [file Image_3.TIF]

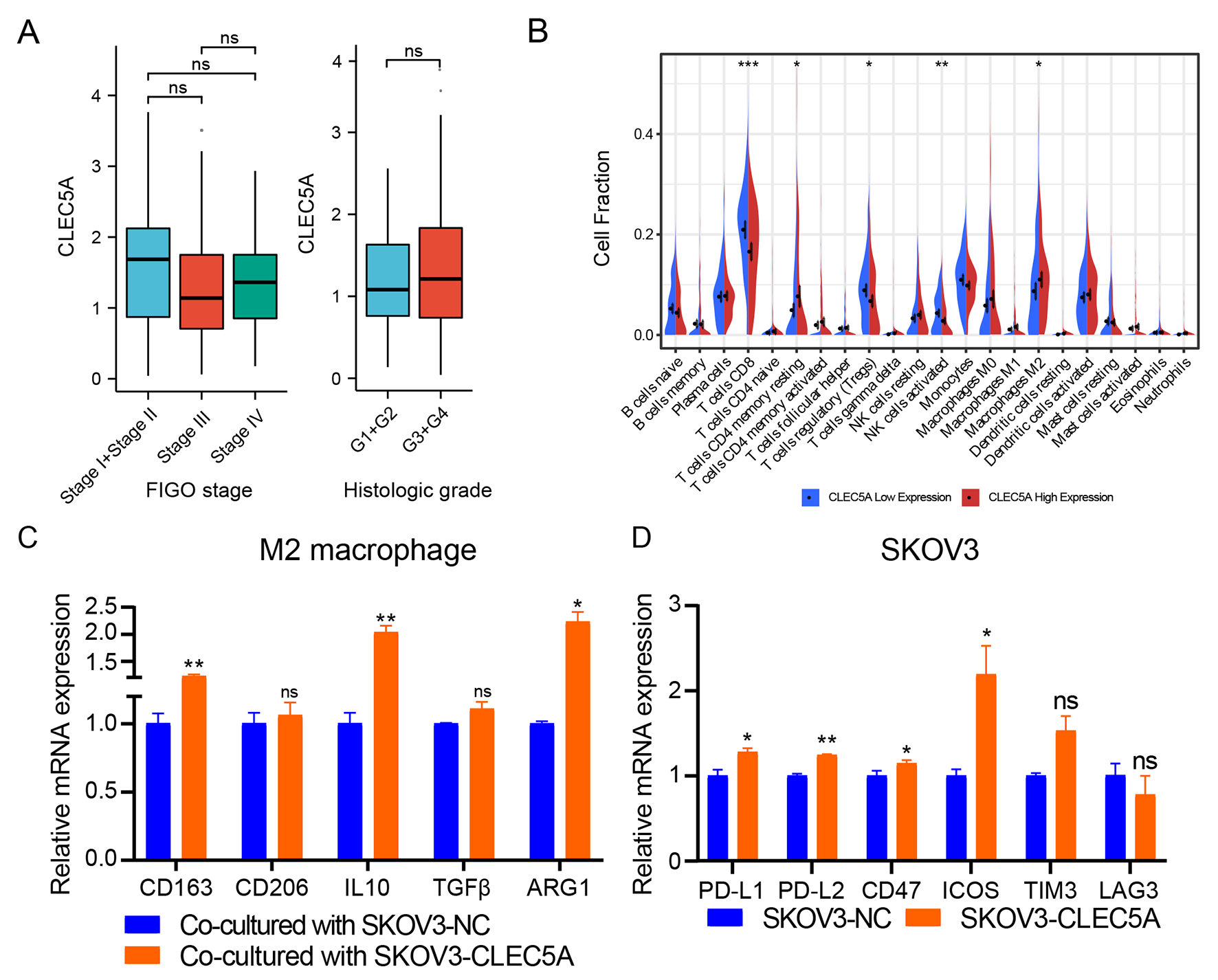

Supplement: Supplementary file 6 [file Image_4.TIF]
